# Case of Accumulation of Fluid in the Ureter and Pelvis of the Kidney, upon the Right Side, of Long Standing; Followed by Retention and Suppression of Urine, Acute Inflammation of the Bladder and Left Kidney, Terminating in Suppuration and Death

**Published:** 1847-10

**Authors:** W. B. Herrick

**Affiliations:** Professor of Anatomy in Rush Medical College


### ARTICLE VI.

*Case of Accumulation of Fluid in the Ureter and Pelvis of the Kidney, upon the right side, of long standing; followed by retention and suppression of urine, acute inflammation of the bladder and left kidney, terminating in suppuration and*

*death.* By W. B. Herrick, M. D., Professor of Anatomy in Rush Medical College.

On the 10th of September I was called, late at night, to see a Mr. L——, who informed me that he had once had an attack of fever attended with tenderness and severe pain in the right lumbar region, and that frequently since then, micturition had been painful and difficult.

At the time I was called, he had had retention of urine for forty-eight hours previous. During this time an unsuccessful attempt had been made, by a physician in the country, to introduce an instrument into the bladder, and what was worse, he had been permitted to ride in a stage coach over rough roads, more than twenty miles, to this city, for the purpose of seeking relief.

I found him, as I anticipated after hearing the above account, suffering most intensely from pain in the pelvic region, with tongue and skin hot and dry, and an irritable pulse ranging from 120 to 130.

By means of a gum elastic catheter, introduced with ease and without giving much pain, about a pint of high colored urine was drawn from the bladder.

This small quantity, the only discharge for forty-eight hours, was by no means equal to the amount usually evacuated after retention that length of time. A fact which led to the conclusion at once, that suppression existed as well as retention.

After this evacuation the pain and other bad symptoms were to some extent relieved, still, however, at intervals of about every ten minutes, spasmodic contractions of the bladder would return, and with them, much of the distress which previously existed.

Diluents, diuretics and anodynes were used freely, together with warm fomentations applied externally to the region of the bladder. But the relief obtained by their use was only temporary, for in a few hours all the inflammatory symptoms returned very much aggravated, and by the morning of the 11th, the pain and tenderness in the region of the bladder had become very much aggravated, the skin and tongue dry, the pulse full, and ranging again from 120 to 130.

These aggravated symptoms were promptly met by a full bleeding to an extent to produce faintness and a weak and soft pulse, and by the administration of cathartics sufficient to produce full and free evacuations, followed by anodynes, diluents, diuretics and warm fomentations as before.

This treatment was followed by a temporary abatement of the fever, and the transitory disappearance of many of the above well marked indications of high inflammatory action in the bladder; but by the morning of the 12th all the general indications of a return of local inflammation made their appearance again, accompanied by nausea and vomiting, pain, fullness and tenderness in the left lumbar region, with scanty and high colored urine. All symptoms indicating that the inflammation had extended to the left kidney, requiring a continuation of active antiphlogistic treatment.

These indications were accordingly met by another full bleeding from the arm and with leeches applied to the left lumbar region, followed by anodynes, diluents, fomentations, &c., with the effect of moderating, again, all the symptoms of acute inflammatory action, and preventing their recurrence subsequently with anything like the same degree of intensity as before.

It was soon found, however, that all efforts to restore their healthy functions to the diseased organs would prove ineffectual, for on visiting my patient the next day I found him much prostrated, with the mental faculties torpid, a black incrustation upon the tongue and teeth, the abdomen tympanitic, no pain except that produced by pressure upon the loins, and a pulse tense and rapid, having frequent and copious discharges of dark bloody matter from the rectum, and an occasional slight evacuation of high colored urine mixed with blood from the bladder. To these symptoms, indicative of the purulent stage of inflammation and of the presence of urea in the blood, were soon added, oppressed respiration and coma. Death followed on the 18th, ten days after the acute attack.

The following were the interesting results of our examination of the body. Upon opening the abdominal cavity and tracing the intestinal canal from above downwards, the mucous membrane of all those portions of the small and large intestines in the vicinity of the left lumbar region, and of the pelvic cavity presented all the appearances consequent upon recent acute inflammation, and in some parts, as the rectum and lower part of the colon, it had extended to all the layers, so as to produce thickening and adhesions of the peritoneum. In tracing the urethra from below upwards through the membranous and prostatic portions, no indications of previous disease, excepting a slight redness of the mucous membrane could be detected, but upon arriving at, and examining the bladder, it was found contracted to one fourth its natural size, and in a position to the left of the median line, with its right external surface in contact with, and adherent to a large fluctuatory tumor, which was found to occupy the whole space, beneath the peritoneum, between it and the walls of the pelvis upon the right side. Upon cutting through its walls, which were thick and muscular, the cavity was found to be small, and the mucous membrane lining it injected and softened. The opening for the ureter of the left side was easily found, but none could be discovered upon the right.

An opening was next made through the walls of the bladder into the tumor, which was found to consist of a large fibrous sack lined by mucous membrane, and filled with about a pint of a dark brown fluid.

Continuous with the walls of this sack, there was a canal, which upon being traced upwards, was found to terminate at the upper part of the right illiac region, in an encysted tumor, composed of indurated glandular structure upon the external side, and of fibrous lined with mucous membrane upon the internal, and of a cavity continuous with that of the canal and the large sack, filled also with the dark brown fluid.

This condition of parts had evidently resulted from an obstruction of long standing, to the passage of urine into the bladder.

Upon examining the kidney of the left side it was found distended, engorged with blood, and filled with numerous small abscesses. These were pathological changes which the symptoms, before death, had led us to anticipate.

The enlargement of this kidney, which was about five times the natural size, was, in part no doubt, due to its having performed for a long time, as was evident from the condition of the other, a double function.

